# Proximal Sensing of Plant-Pathogen Interactions in Spring Barley with Three Fluorescence Techniques

**DOI:** 10.3390/s140611135

**Published:** 2014-06-24

**Authors:** Georg Leufen, Georg Noga, Mauricio Hunsche

**Affiliations:** Department of Horticultural Science, Institute of Crop Science and Resource Conservation, University of Bonn, Auf dem Huegel 6, Bonn D-53121, Germany; E-Mails: gleufen@uni-bonn.de (G.L.); noga@uni-bonn.de (G.N.)

**Keywords:** chlorophyll fluorescence, blue-green fluorescence, *Blumeria graminis*, *Puccinia hordei*, fluorescence imaging, fluorescence lifetime

## Abstract

In the last years fluorescence spectroscopy has come to be viewed as an essential approach in key research fields of applied plant sciences. However, the quantity and particularly the quality of information produced by different equipment might vary considerably. In this study we investigate the potential of three optical devices for the proximal sensing of plant-pathogen interactions in four genotypes of spring barley. For this purpose, the fluorescence lifetime, the image-resolved multispectral fluorescence and selected indices of a portable multiparametric fluorescence device were recorded at 3, 6, and 9 days after inoculation (dai) from healthy leaves as well as from leaves inoculated with powdery mildew (*Blumeria graminis*) or leaf rust (*Puccinia hordei*). Genotype-specific responses to pathogen infections were revealed already at 3 dai by higher fluorescence mean lifetimes in the spectral range from 410 to 560 nm in the less susceptible varieties. Noticeable pathogen-induced modifications were also revealed by the ‘Blue-to-Far-Red Fluorescence Ratio’ and the ‘Simple Fluorescence Ratio’. Particularly in the susceptible varieties the differences became more evident in the time-course of the experiment *i.e.*, following the pathogen development. The relevance of the blue and green fluorescence to exploit the plant-pathogen interaction was demonstrated by the multispectral fluorescence imaging system. As shown, mildewed leaves were characterized by exceptionally high blue fluorescence, contrasting the values observed in rust inoculated leaves. Further, we confirm that the intensity of green fluorescence depends on the pathogen infection and the stage of disease development; this information might allow a differentiation of both diseases. Moreover, our results demonstrate that the detection area might influence the quality of the information, although it had a minor impact only in the current study. Finally, we highlight the relevance of different excitation-emission channels to better understand and evaluate plant-physiological alterations due to pathogen infections.

## 1. Introduction

During their whole lifecycle, agricultural crops are exposed to a multitude of harmful organisms *i.e.*, pathogens, that cause considerable yield losses. Obligate biotrophic parasites, like powdery mildew and leaf rust, cause the most serious and widespread diseases in agronomic crops [1]. Despite crop protection activities, estimations indicate that more than 10 per cent of the worldwide wheat losses can be attributed to pathogens [2]. The adoption of varieties which are resistant to the pathogens is one promising and environment-friendly attempt to mitigate this problem. Nevertheless, the development of new varieties in traditional breeding programs is an expensive and time-consuming process, and requires many expensive field studies and validations over several years [3]. In breeding programs new and promising lines are classified and rated to their susceptibility to diseases after a visual monitoring done by trained specialists. However, precise classifications on the susceptibility to pathogens are often difficult, amongst others due to interpretations of the operators [4].

In recent studies, the potential of non-invasive techniques for the detection of plant diseases was demonstrated [5,6]. In particular, chlorophyll fluorescence (ChlF) could be adopted as a reliable tool to estimate plant responses to different types of pathogens [7,8]. On wheat leaves, pathogen attacks raise the ChlF Red/Far-Red ratio, indicating photosynthetic impairments and a possible decrease of chlorophyll content [9]. Besides the ChlF, which is primary emitted by chlorophyll *a* molecules [10], several phenolic substances and other fluorophores emit a characteristically blue (F440) or green (F530) fluorescence when excited with UV radiation [11,12]. Bürling *et al.* [9,13,14] have highlighted the potential of selected fluorescence ratios, such as the blue-to-green (F451/F522), the blue-to-red (F451/F687) and the blue-to-far-red ratio (F451/F736) for a pre-symptomatic or at least early detection of powdery mildew and leaf rust in susceptible and resistant wheat varieties. An alternative to these well-established fluorescence ratios is the determination of fluorescence lifetime [12]. Modifications in the lifetime, as a result of pathogen infection, might result from the accumulation of defence-related secondary compounds leading to longer fluorescence decay [13,14]. Substances like salicylic acid and phenylpropanoid compounds were previously identified as key ones in plant disease resistance [15].

In the last decades significant advances were made in understanding the *in vivo* and *in situ* pigment fluorescence, and the relevance of the several influencing factors for the quality and reliability of the results. Despite the promising perspectives for applied research in plant sciences and practical use in agriculture [12], extensive agronomic and phytopathological studies aiming to explore the potential of different types of fluorosensing devices are still scarce.

The studies done by Bürling *et al.* [9,13,14] served as a basis for our current work targeting the potential of the imaging-based spectrally resolved fluorescence and fluorescence-indices of a portable multiparametric device to assess the impact of powdery mildew (*Blumeria graminis* f. sp. *hordei*) and leaf rust (*Puccinia hordei*) on the fluorescence signature of four spring barley varieties. In this context we aimed a better understanding concerning the detection area and the excitation light for the assessment of plant-pathogen interactions. Moreover, we hypothesized that specific fluorescence indices would enable the characterization and differentiation genotype-specific responses to the diseases. With this background, we set the experiments under controlled conditions using four spring barley varieties with different susceptibility degrees to powdery mildew and leaf rust. Fluorescence lifetime, image-based spectrally resolved fluorescence intensity and several fluorescence-indices of a handheld sensor were recorded from a pre-symptomatic stage (3 days after inoculation, dai) until the stage where strong disease symptoms became visible (9 dai).

## 2. Experimental Section

### 2.1. Plant Material and Growth Conditions

Experiments were conducted sequentially in environment-controlled growth cabinets simulating a 16 h photoperiod with 150 μmol·m^−^^2^·s^−^^1^ photosynthetic active radiation (PAR; Philips PL-L 36W, Hamburg, Germany), a day/night temperature of 20/18 ± 2 °C and a relative humidity of 70/80 ± 5%. The spring barley (*Hordeum vulgare* L.) varieties Belana and Marthe (Saaten Union GmbH, Isernhagen, Germany) and Conchita and Tocada (KWS Saat AG, Einbeck, Germany), differing in their susceptibility degree (SD) to powdery mildew and leaf rust (Table 1), were selected for the experiments. Untreated seeds were sown into 0.27 l plastic pots (0.08 m height, 0.07 m diameter), evenly filed with commercial peat substrate (Einheitserde Typ VM, Einheitserde- und Humuswerke Gebr. Patzer GmbH & Co.KG, Sinntal-Altengronau, Germany). Plants were regularly watered with tap water. One week after germination, seedlings were thinned out to maintain one plant per pot. Plants (*n =* 5 per genotype and treatment) were placed at random into the growth chambers. Twenty-one days after sowing the inoculation of the pathogens was performed at the second fully expanded leaf (BBCH stage 12).

### 2.2. Inoculation of Puccinia hordei and Blumeria graminis f. sp. hordei

The inoculation of *Puccinia hordei* was done according to the method described by Bürling *et al.* [13], with minor modifications. Briefly, *Puccinia hordei* spores (courtesy of the Department of Phytomedicine, University of Bonn, Bonn, Germany) were suspended in a solution of distilled water and Tween 20 (0.01%, Merck-Schuchardt, Hohenbrunn, Germany). After estimating the spore concentration with a Fuchs-Rosenthal counting chamber, the spore density was adjusted to 3.8 × 10^4^ spores·mL^−^^1^. Afterwards, leaves were fixed horizontally on a sample holder and twelve 6 μL droplets of spore suspension were evenly distributed on the adaxial side, starting at seven centimetres downwards from the leaf tip. Thereby, the inoculated area (approx. 4.5 cm^2^) was labelled with a felt tip pen for the subsequent fluorescence determinations. Plants were kept for 24 h into a closed environment with a relative humidity ≥95% to provide optimum environment for spore germination and the establishment of disease. Control plants were handled in similar way, but were treated with droplets of distilled water and Tween 20 (0.01%) only.

The inoculation of *Blumeria graminis* followed the method described in the literature [14]. Thereby, conidia of powdery mildew (Department of Phytomedicine, University of Bonn) were removed with a fine brush from infected plants and evenly distributed over the whole adaxial leaf surface of the experimental plants, particularly in a section of seven centimetres from the leaf tip. The inoculated area was marked by felt tip pen; twenty-four hours after inoculation visible conidia were removed by gently blowing over the leaf surface.

### 2.3. Fluorescence Measurements

Fluorescence measurements were conducted at leaf level by using a compact fiber-optic spectrometer (IOM GmbH, Berlin, Germany), a multispectral fluorescence imaging system (Nuance TM^FX^, Caliper Life Sciences, PerkinElmer, MA, USA) and a hand-held optical fluorescence technique (Multiplex3^®^, Force-A, Orsay, France). As important characteristic, the size of detection area was significantly different between the used methods ranging from approximately 1 mm^2^ (laser fluoroscope used for lifetime recordings) to 40 cm^2^ (portable equipment to record multiple fluorescence indices), as shown in the Supplemental Material (Figure S1). With exception of the fluorescence images which were taken under dark conditions, fluorescence recordings were done in the lab (average temperature 21 °C) under ambient light. Spectroscopic analysis of leaves inoculated with powdery mildew and leaf rust were done separately in two consecutive phases to avoid multiple stress caused by both pathogens.

#### 2.3.1. Fluorescence Lifetime

Settings and instrumental setups of the laser spectrometer were similar as described elsewhere [14]. In our experiments the fiber-optic spectrometer was used to record the fluorescence lifetime in in a range of 410 to 560 nm with an interval of 30 nm. For this purpose a pulsed nitrogen laser (MNL 100, LTB Lasertechnik Berlin GmbH, Berlin, Germany) with excitation at 337 nm and repetition rate of 30 Hz was used. The pulse energy at the probe exit was adjusted to be 2–3.5 μJ. A photomultiplier (PMT, H5783-01, Hamamatsu, Japan), with a sensitivity of 800 Volt, was used as detector. The detection gate was opened from 0.0 to 16 ns following excitation, and the step width of the integrator gate was set to 0.4 ns. Each single data point was calculated by an average of 16 pulse counts. Before measuring, leaves were placed on a horizontal sample holder by keeping a constant distance (3.95 mm) between sample and the optical probe. Fluorescence decay was analyzed by using deconvolution software (DC4, V. 2.0.6.3, IOM GmbH, Berlin, Germany). Fluorescence lifetime readings were taken from control and pathogen inoculated leaves eight centimetres from the leaf tip. Particularly on leaf rust inoculated leaves, efforts were made to ensure that readings were always taken over the inoculated area.

#### 2.3.2. Fluorescence Imaging

Fluorescence images were recorded with a 1.4 megapixel CCD camera mounted onto a stereomicroscope (Zeiss Stereo Lumar V12, Jena, Germany). Three Zeiss Lumar filters (01, 09 and 14) enabled the fluorescence excitation in spectral ranges about 365 ± 12 nm (UV), 450–490 nm (blue) and 510–560 nm (green). Fluorescence data were acquired using a 0.8 X Zeiss Neo Lumar objective. A mercury short-arc lamp (HXP R 120W/45C UV, Osram, München, Germany) installed into a cold-light (LQ-HXP 120, Leistungszentrum Jena, Jena, Germany), was used as illumination source. To ensure clear images with a high data quality, images had to be recorded by using the highest light intensity, reaching 111,170 μW·cm^−^^2^ (UV filter), 128,882 μW·cm^−^^2^ (blue filter), and 30,346 μW·cm^−^^2^ (green filter) at leaf level. Leaves were fixed on a specially developed sample holder; here, a vacuum device produces a controllable negative pressure so that leaves lay flat on the surface. Settings were adjusted by 11x magnification and a focus of 51.4 mm to evaluate an object field of 110.25 mm^2^. Fluorescence intensities were recorded under different excitation light sources in 10 nm steps for the following spectral ranges: 420–500 nm (blue), 500–580 nm (green) and from 620–720 (red) nm; signals were detected where at least 100 adjacent pixels had the same signature. Exposure time was automatically defined for each sample. Finally, the images were analyzed by using Nuance 2.4 imaging software. This software performs an automatic unmixing of fluorescence intensities and enables the determination of the corresponding fluorescing area. To increase the data quality, a spectral library was created for each excitation/emission range of control and pathogen inoculated varieties at 3, 6 and 9 dai.

#### 2.3.3. Portable Multiparametric Fluorescence Sensor

The hand-held fluorescence technique enables to record multiple fluorescence indices [16] to sense the response of plants to environmental factors under semi-controlled [17] and field conditions [18]. Briefly, light-emitting-diodes excite the fluorescence with UV excitation (peak at 375 nm), green light (peak at 518 nm) and red light (peak at 630 nm) while the emitted fluorescence is detected in the blue (425–475 nm), red (680–690 nm) and far-red (720–755 nm) spectral region. Recordings were conducted at leaf level; here, the area of approximately 12.56 cm^2^ was illuminated by maintaining a constant distance of 0.10 m between sensor and leaf surface. In a preliminary screening, the ‘Simple Fluorescence Ratio’ (SFR) and the ‘Blue-to Far-Red Fluorescence Ratio’ (BFRR_UV) yielded the most promising results to sense both fungal diseases. The SFR is the inverse fluorescence ratio of the chlorophyll fluorescence ratio F680/F730 recorded with green excitation, whereas the BFRR depends on the blue and far-red fluorescence, recorded with UV excitation.

### 2.4. Statistical Analysis

Data were statistically analyzed with SPSS statistic software (PASW statistics version 19.0, SPSS Inc., Chicago, IL, USA). For each genotype, pathogen and evaluation date, means of five control and five pathogen inoculated plants were compared by analysis of variance and paired *t*-test (*p ≤* 0.05).

## 3. Results

### 3.1. Fluorescence Lifetime

**Figure 1.**
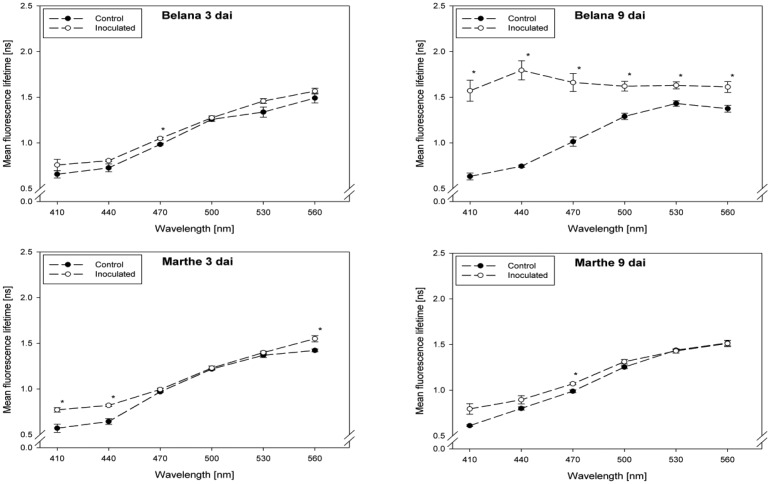

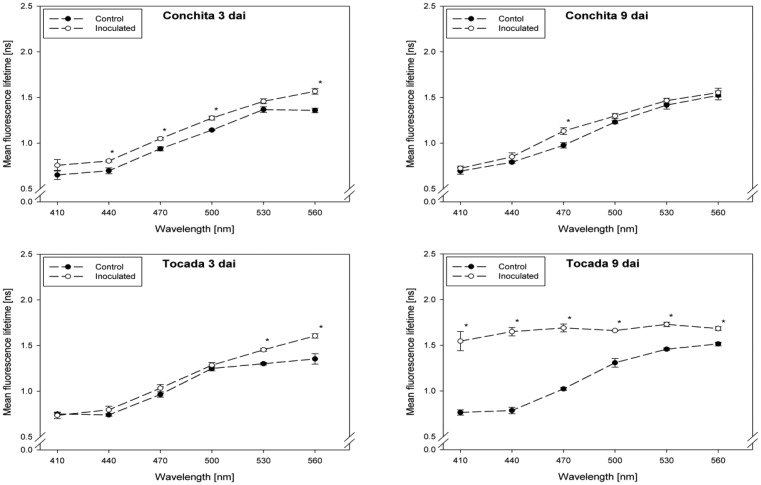
Mean fluorescence lifetime at selected wavelength (410–560 nm) recorded from control and powdery mildewed leaves of the barley varieties Belana, Marthe, Conchita and Tocada (from top to bottom) at 3 and 9 days after inoculation (dai). Values indicate mean ± standard error (*n =* 5). Significant differences (*t*-test *, *p ≤* 0.05) between control and inoculated leaves for each variety, wavelength, and measuring day are shown.

**Figure 2.**
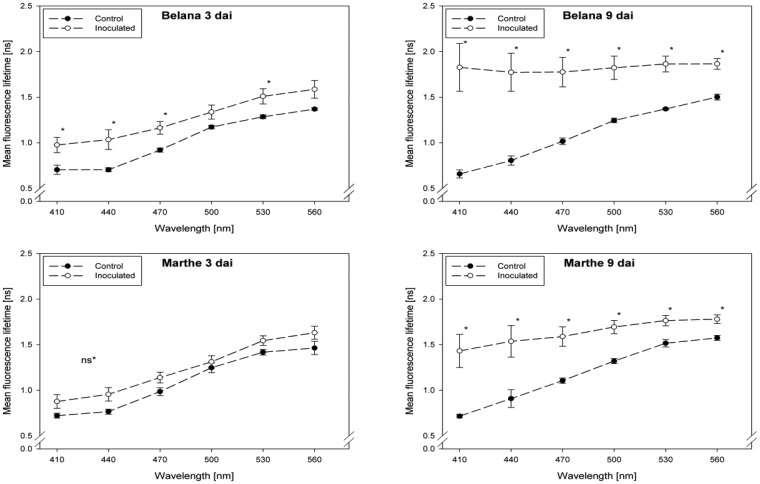

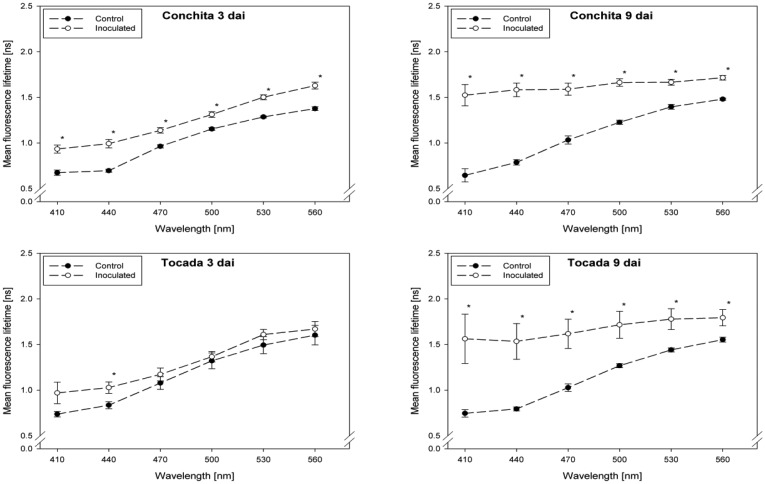
Mean fluorescence lifetime at selected wavelength (410–560 nm) of control and leaf rust inoculated leaves of the barley varieties Belana, Marthe, Conchita and Tocada (from top to bottom) at 3 and 9 days after inoculation (dai). Values indicate mean ± standard error (*n =* 5). Significant differences (*t*-test *, *p ≤* 0.05) between control and inoculated leaves for each variety, wavelength, and measuring day are shown.

Fluorescence lifetime in healthy and inoculated leaves at 410, 440, 470, 500, 530 and 560 nm displayed in Figures 1 and 2. The impact of powdery mildew (Figure 1) and leaf rust (Figure 2) is shown for each variety on the third and ninth day after inoculation. In the most cases, inoculation of the leaves led to higher mean fluorescence lifetime as compared to the control (healthy) leaves, but each situation (combination of variety, pathogen, dai, wavelength) has to be considered separately.

A detailed analysis indicate that ‘Conchita’ had the most pronounced differences between inoculated and control plants at 3 dai at the wavelengths 560 nm, followed by 500, 470 and 440 nm (Figure 1). At the same time, significant differences between healthy and diseased leaves were ascertained for the varieties Belana (470 nm), Marthe (410, 440 and 560 nm) and Tocada (530 and 560 nm). Recordings at 6 dai *(data not shown*) and 9 dai indicate that mean fluorescence lifetime in ‘Conchita’ and ‘Marthe’ had a similar pattern as observed at 3 dai. In ‘Tocada’ significant differences were observed at 6 dai particularly in the spectral range from 440 to 530 nm (not shown). At 9 dai, fluorescence lifetime in the range of 410–560 nm was significantly higher in the diseased tissues of both ‘Belana’ and ‘Tocada’, whereas ‘Marthe’ and ‘Conchita’ exhibited only slight alterations at 470 nm (Figure 1).

In general, inoculation of plants with leaf rust raised the fluorescence mean lifetime in all varieties and wavelengths, even if not always statistically significant. Evaluations at 3 dai indicate that ‘Marthe’ and ‘Tocada’ were less affected while ‘Belana’ and ‘Conchita’ were more sensitive by leaf rust (Figure 2). At 6 dai (data not shown) significant differences between both treatment groups, control and inoculated leaves, were determined in the four varieties nearly in all wavelengths. Thereby, the numerical difference between healthy and diseased leaves was more pronounced in the blue spectral range between 410 and 470 nm. This trend was confirmed at the end of the experiment (9 dai), when leaves infected with rust caused a strong increase of mean fluorescence lifetime in comparison to the control plants, but also to the previous evaluation dates.

### 3.2. Fluorescence Images

#### 3.2.1. Indications Provided by Selected Spectral Ranges

Divided into three spectral ranges (420–500 nm, 500–580 nm, 620–720 nm), we recorded the fluorescence intensity from 420 to 720 nm by using a spectral camera with different excitation sources. To enable a fast and precise overview of our major findings we summarize the outcomes in a simplified manner by indicating the significant differences between control and inoculated plants (Table 2). With exception of ‘Belana’ at 6 dai, powdery mildew inoculation led to significant differences between control and mildewed leaves in the UV excited blue fluorescence (420–500 nm) in all varieties (Table 2). This trend was also observed for the blue excited green fluorescence (500–580 nm), irrespective of the susceptibility degree. Differently, leaf rust led to more frequent and pronounced differences at 6 and 9 dai. In general, both diseases caused only minor changes in the ChlF (620–720 nm). However, green induced ChlF responded quite sensitively to pathogen infection (Table 2). Particularly the mildewed leaves of ‘Marthe’ were characterized by a significant higher ChlF (data not shown).

#### 3.2.2. Green Fluorescence Intensity

**Figure 3.**
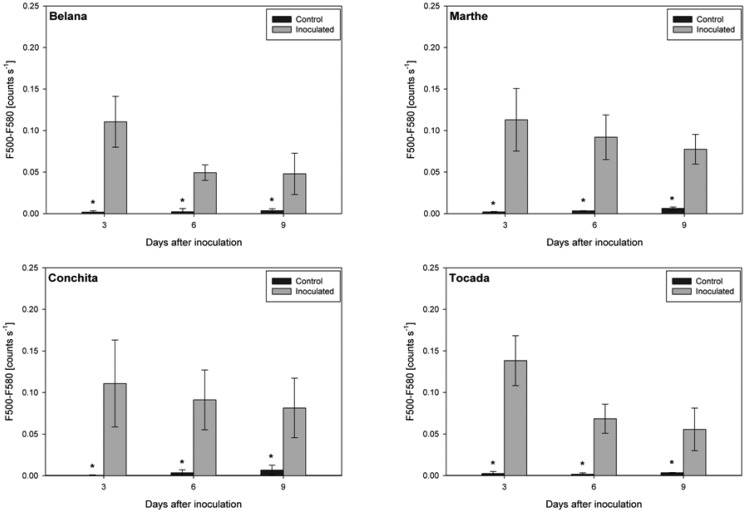
Green fluorescence intensity (500–580 nm scaled as counts s^−^^1^) recorded under blue excitation. Leaves of the healthy control and powdery mildewed plants of the barley varieties Belana, Marthe, Conchita and Tocada were studied at 3, 6, 9 days after inoculation. Values indicate mean ± standard error (*n =* 5). Asterisk (*) indicate significant differences (*t*-test, *p ≤* 0.05) between control and inoculated leaves for each variety and measuring day.

Irrespective of variety and measuring day, the blue excited green fluorescence in powdery mildewed leaves was significantly higher as compared to the respective control plants (Figure 3). Moreover, in the time-course from 3 to 9 dai, green fluorescence intensity in powdery mildewed leaves of ‘Belana’ and ‘Tocada’ decreased to a larger extent than in ‘Marthe’ and ‘Conchita’.

**Figure 4.**
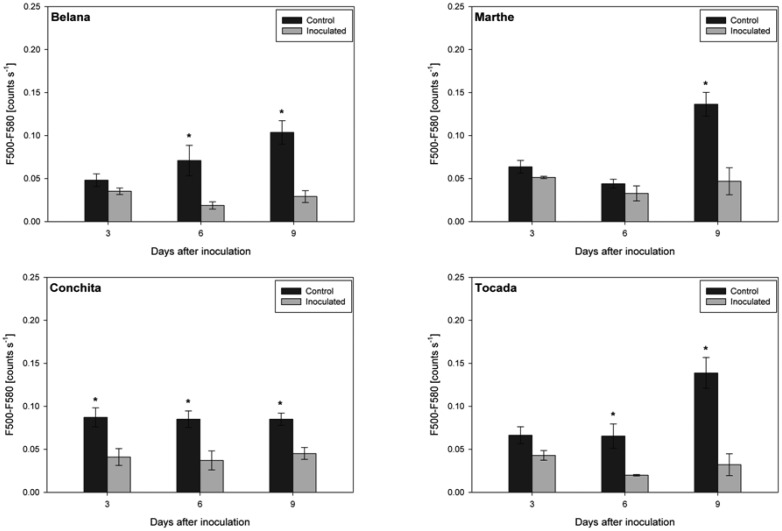
Green fluorescence intensity (500–580 nm scaled as counts s^−^^1^) recorded under blue excitation. Leaves of the healthy control and leaf rust inoculated plants, of the barley varieties Belana, Marthe, Conchita and Tocada were studied at 3, 6, 9 days after inoculation. Values indicate mean ± standard error (*n =* 5). Asterisks (*) indicate significant differences (*t*-test, *p ≤* 0.05) between control and inoculated leaves for each variety and measuring day.

In contrast, leaves inoculated with leaf rust had distinctly lower green fluorescence intensity as compared to control leaves (Figure 4). Thereby, ‘Conchita’ displayed strong differences between control and inoculated leaves from 3 to 9 dai. Differences of lower magnitude were observed for ‘Belana’ and ‘Tocada’ (6 and 9 dai) as well as ‘Marthe’ at 9 dai.

#### 3.2.3. Green Fluorescence Intensity of Infected Leaf Area

Due to strong effect of the pathogen inoculation on the blue excited green fluorescence (500–580 nm), we calculated the leaf area with similar emission properties in order to identify the area of the tissue affected by the pathogens. In the time-course from 3 to 9 dai, the area of green fluorescence intensity in powdery mildewed leaves of ‘Belana’ and ‘Tocada’ increased to a larger extent than in ‘Marthe’ and ‘Conchita’. As shown, we recorded significant differences between control and powdery mildewed leaves for all varieties and measuring days (Table 3). Similar results were obtained in the leaf rust study; however, here differences were only observed in ‘Marthe’ and ‘Conchita’ at 3 and 6 dai (Table 3).

### 3.3. ‘Blue-to-Far-Red Fluorescence’ and ‘Simple Fluorescence Ratio’

Powdery mildew led to significant alterations of the Blue-to-Far-Red Fluorescence Ratio (BFRR_UV) and the ‘Simple Fluorescence Ratio’ (SFR_G) in the varieties Belana and Tocada (Table 4).

Thereby, a considerable increase of BFRR_UV could be determined in the inoculated leaves of ‘Belana’ and ‘Tocada’ while no differences between control and inoculated leaves were observed for both SFR_G and BFRR_UV on ‘Marthe’ and ‘Conchita’. On the other hand, leaf rust inoculation significantly changed the BFRR_UV in inoculated leaves of ‘Belana’ and ‘Tocada’ but not of ‘Marthe’ and ‘Conchita’ (Table 4). In addition, at 3 and 6 dai the SFR_G displayed significant differences between control and inoculated leaves in ‘Belana’ and ‘Marthe’, and for ‘Tocada’ at 6 and 9 dai. In contrast, no differences were found for ‘Conchita’.

## 4. Discussion

Our results demonstrate the potential of three techniques - the fluorescence lifetime, the image-resolved multispectral fluorescence and selected indices of a portable multiparametric fluorescence sensor-for the proximal sensing of plant-pathogen interactions in spring barley. Irrespective of the remarkable technical differences between the sensors, in particular with respect to the analysed area and the spectral characteristics for excitation and detection, the fluorescence devices used here enabled to sense the impact of powdery mildew and leaf rust, and indicated some genotype-specific responses to these pathogens. The selected fluorescence signals and indices reflect changes in the amount and chemical composition of different compounds and substance groups including chlorophyll *a* and *b*, plant polyphenols, and pathogen-originated fluorophores.

In our studies we adopted a commercially available fluorescence imaging system originally developed to screen the efficacy of medicinal products in the pharmaceutical industry (Figure S1C). Similar to the findings of Rousseau *et al.* [19] and Pineda *et al.* [20], our studies showed temporal and spatial changes in the fluorescence of control and pathogen inoculated leaves. Thereby, *Blumeria graminis* f. sp. *hordei* and *Puccinia hordei* caused only minor alterations in the ChlF as compared to the BGF (Table 2); this can be attributed to the biotrophic relationship of the pathogens with their host [21]. As shown, both foliar diseases led to variations in the UV-excited blue (420–500 nm) as well as in the UV and blue excited green fluorescence (500–580 nm). With this technique, which is designed for operation in the laboratory under dark conditions, it is possible to differentiate the impact of both leaf diseases (Table 2). In particular, powdery mildew significantly influenced the UV-excited blue fluorescence, irrespective of the susceptibility degree of the genotypes (Table 2). While control leaves displayed a characteristic blue fluorescence which mainly originates from trichomes and/or leaf veins [22], the higher values recorded on mildewed leaves arise from the blue fluorescing inoculum, e.g., conidiophores (Figure S2). Residues of the inoculum as well as newly formed fungal structures overlap and partially shield the plants' natural fluorescence. In this context our findings confirm previous observations [23] suggesting that the development cycle of other obligate biotrophs is accompanied by a characteristically blue autofluorescence.

As reported by Lüdeker *et al.* [4], fungal infection led to a stronger increase in the green (F520) than in the blue fluorescence (F440), that can be either caused by the fungi or due to accumulation of intercellular substances. According to our results, the inoculum of *Blumeria graminis* f.sp. *hordei* exhibits a typical blue-green fluorescence under UV excitation, whereas spores of *Puccinia hordei* produce a rather green-orange spectral range. On barley leaves, blue excitation caused a significantly rise of the fluorescence intensities, and on powdery mildew spores to a stronger shift towards the green-orange spectral range (Figure S2). Spore specific fluorescence patterns might explain why the blue excited green fluorescence was the most useful parameter to identify the temporal and spatial development of both diseases (Table 2).

Differences concerning the inoculation method might explain the strong variations in the green fluorescence intensity when comparing both pathogens. Powdery mildew spores were inoculated across the leaf surface, whereas rust spores were spot inoculated by placing droplets of a spore suspension. After inoculation, green fluorescence intensity measured on the powdery mildew resistant varieties Marthe and Conchita dropped slower as compared to the susceptible varieties Belana and Tocada. Two processes might have contributed for these results: firstly, the mycelium at the surface of susceptible varieties changed the optical properties in the time course of our study; secondly, the reduced ability of susceptible varieties to overcome the pathogen attack. Increasing area of green fluorescence intensity in the time course of our study supports our first assumption (Table 3). Moreover, minor changes in the green fluorescence intensity of the resistant varieties suggest the accumulation of pathogen or resistance specific compounds, such as lignin, and/or the production of waxes, affecting the fluorescence emission [24–28]. To this point our findings confirm and support previous studies which focussed on the process of fungal influencing the autofluorescence of leaves [29], even if we did not record the fluorescence of single cells. A completely different trend was shown in rust inoculated leaves which exhibited a significantly lower green fluorescence as their respective control leaves. Control leaves might undergo a significantly faster aging resulting in a higher blue-green fluorescence [30], whereas rust infection can cause a delay of normal senescence, due to an increase in the concentration of cytokinins [31].

Differently than the time-consuming spectroscopic technique used by Bürling *et al.* [13,14], we assessed the most promising fluorescence ratios (SFR_G and BFRR_UV) with a significantly faster operating hand-held fluorometer. Leaves inoculated with *Blumeria graminis* f. sp. *hordei* demonstrate a first increase after pathogen inoculation (3 dai), which was followed by a pronounced decrease (6–9 dai) of the ChlF-ratio F730/F685, here referred as SFR_G (Table 4). These changes were more pronounced in the susceptible varieties Belana and Tocada, as compared to the resistant varieties Conchita and Marthe. Similar results are reported in the literature [7,32]. Modifications of the SFR_G were caused by a first disruption of the photosynthetic quantum conversion and consequently in a later decrease of the chlorophyll content [33]. Leaf rust causing punctual diseased spots significantly influenced the chlorophyll fluorescence (displayed as SFR_G) in all four varieties (Table 4). Similar results were reported by Scholes and Rolfe [8] and Bürling *et al.* [34], who showed that the photosynthesis of regions which were not invaded by the fungal mycelium was severely impaired. The analogous trend observed for all varieties is explained amongst others by their comparable susceptibility degree in the range from 4 to 6 (Table 1). In this context, either the higher susceptibility degrees of the four barley varieties to powdery mildew or different patterns in the infection and development cycles of mildew and rust, might explain the immediate decline of SFR_G values in rust infected leaves at 3 dai. While *Blumeria graminis* f. sp. *hordei* exclusively affects the epidermal cell layer [35], *Puccinia hordei* infects also mesophyll cells and modifies the chloroplasts [36]. Contrasting the limited potential of the ChlF recorded with the imaging system, the extensive results recorded with the hand-held sensor can be explained by its wider spectral range which covers completely both chlorophyll emission ranges. Both fungal diseases were also indicated by BFRR_UV index. In case of powdery mildewed leaves, BFRR_UV followed the same trend as described for SFR_G, indicating that these changes were mainly caused by chlorophyll degradation [37,38]. Comparable results were ascertained by Lüdeker *et al.* [4] on rust infected wheat leaves. Finally, both foliar diseases led to a considerable increase in the mean fluorescence lifetime. Due to their comparable susceptibility degree to leaf rust, the results observed for the four varieties followed the same trend. Nevertheless, at 3 dai ‘Belana’ and ‘Conchita’ (less susceptible to rust) exhibit a significant higher mean lifetime from 410–560 nm than ‘Marthe’ and ‘Tocada’ (higher susceptibility). In case of powdery mildew, we confirmed previous observations of Bürling *et al.* [14]. Thereby, we indicate higher mean lifetime starting from 500 nm (3 dai) and a later (6–9 dai) rise in the spectral region of 410–500 nm; these results correlate with the appearance of disease symptoms and the strong blue fluorescence intensity of *Blumeria graminis* spores (Figure S2).

Compared to previous studies on the impact of pathogens on the laser-induced spectrally resolved fluorescence [9,13,14], our findings show two significant improvements. Firstly, we did not work only with UV-excitation to assess BGF and ChlF, but we highlight the benefits of multiple fluorescence excitation as essential tool basic and applied studies (Table 2). Secondly, we adopted and tested techniques which are appropriate for different situations, starting with the highly time-resolved fluorescence spectroscopy (punctual measurement with micrometric scale), the spectrally-resolved imaging system (measurements on square centimetre level), and a robust equipment suitable for field evaluations (Figures S1). Besides the wide range concerning the detection area, we show that all tested methods enable the detection of spectral modifications caused by leaf diseases. In addition to the system-specific excitation and the spectral detection ranges, there are other pertinent differences which influence the efficiency of these methods for larger scientific studies or practical applications. Up to the hand-held fluorescence device, the other techniques can only be run in the laboratory, sometimes requiring extensive preparation time before and after the fluorescence readings. Changes in the amount and or composition of fluorophores might be better recorded with the laser-induced fluorescence spectrometer. Visualization of dynamic plant-pathogen interactions, as shown with our high-resolution fluorescence camera, is also not possible with the field-suitable technique. At this point we see the main benefit of imaging methods since foliar diseases differ in their development cycle and appearance [39], also influencing the spectral signature [5]. To the best of our knowledge, this is the first study where fluorescence images were automatically analysed based on a previously created spectral library. Here, we recognise promising perspectives if a broad spectral library comprising several pathogens, plants, and development stages can be set. If successful, the range of possible assignments include pre-breeding programs and physiological studies in different pathogen-host systems.

## 5. Conclusions

The fluorescence techniques adopted in our studies enabled the detection of pathogen infection and disease development in barley. Susceptible and resistant varieties inoculated with pathogens showed distinct modifications in mean lifetime from 410 to 470 nm, as well in the indices SFR_G and the BFFR_UV. Following the modification of these parameters in the time-course of the experiment it was possible to differentiate the varieties according to their susceptibility degree. The used multispectral fluorescence imaging system provides basic information to distinguish between both diseases, since powdery mildewed leaves significantly exhibit a higher blue and green fluorescence intensity as leaf rust diseased leaves. Finally, we highlight the importance of different excitation and emission ranges for sensing and differentiation of diseases as well as the screening for tolerant and susceptible genotypes. The UV-exited blue fluorescence and the blue-excited green fluorescence offer the most promising information for further studies on these topics.

## Figures and Tables

**Table 1.** Susceptibility degree of the selected varieties to powdery mildew and leaf rust. Classification follows the descriptive variety list of the German Federal Plant Variety Office 2013 in a scale ranging from 1–9 (from less to more susceptible), 5 represents medium susceptibility.

**Susceptibility Degree Against**	**Belana (*Saaten Union*)**	**Marthe (*Saaten Union*)**	**Conchita (*KWS*)**	**Tocada (*KWS*)**
Powdery mildew	6	2	2	7
Leaf rust	4	5	4	6

**Table 2.** Temporal development of the blue (420–500 nm), green (500–580 nm) and ChlF (620–720 nm) for mildewed and rust infected spring barley leaves of the varieties Belana, Marthe, Conchita and Tocada. (x) indicates significant differences between control and pathogen inoculated leaves (*t*-test, *p ≤* 0.05).

**Barley Variety**	**420–500 nm**			**500–580 nm**						**620–720 nm**								
		
	**UV Excited**			**UV Excited**			**Blue Excited**			**UV Excited**			**Blue Excited**			**Green Excited**		
					
	**3dai**	**6dai**	**9dai**	**3dai**	**6dai**	**9dai**	**3dai**	**6dai**	**9dai**	**3dai**	**6dai**	**9dai**	**3dai**	**6dai**	**9dai**	**3dai**	**6dai**	**9dai**
**Powdery mildew**																		

**Belana**	x		x	x			x	x	x									x
**Marthe**	x	x	x	x		x	x	x	x			x		x	x	x	x	x
**Conchita**	x	x	x				x	x	x				x					
**Tocada**	x	x	x	x			x	x	x									x

**Leaf rust**																		

**Belana**					x			x	x	x						x		
**Marthe**	x		x			x			x								x	x
**Conchita**							x	x	x				x	x				
**Tocada**				x	x	x		x	x							x		

**Table 3.** Area of green fluorescence intensity (mm^2^) of control (C), powdery mildew or leaf rust inoculated (I) barley leaves of the varieties Belana, Marthe, Conchita and Tocada at 3, 6 and 9 days after inoculation (dai).

**Barley variety**	**3 dai**		**6 dai**		**9 dai**	
		
	**C**	**I**	**C**	**I**	**C**	**I**
**Powdery Mildew**						

Belana	0.05 ± 0.05 *	15.88 ± 5.58	0.28 ± 0.48 *	26.92 ± 6.03	0.85 ± 1.30 *	37.29 ± 4.78
Marthe	0.07 ± 0.02 *	18.65 ± 5.57	0.14 ± 0.02 *	19.14 ± 5.88	0.98 ± 0.62 *	19.51 ± 5.98
Conchita	0.01 ± 0.02 *	20.58 ± 5.93	0.30 ± 0.35 *	19.67 ± 5.97	0.44 ± 0.38 *	19.97 ± 6.00
Tocada	0.09 ± 0.10 *	17.08 ± 3.75	0.11 ± 0.09 *	27.27 ± 4.94	0.12 ± 0.00 *	37.72 ± 3.52

**Leaf Rust**						

Belana	1.95 ± 0.18 *	13.31 ± 1.09	2.99 ± 1.07 *	23.89 ± 1.88	21.55 ± 1.09 *	28.68 ± 0.80
Marthe	3.19 ± 0.77 *	12.60 ± 0.98	2.30 ± 0.50 *	16.70 ± 3.02	17.47 ± 2.74	20.95 ± 2.09
Conchita	3.76 ± 0.80 *	15.25 ± 2.26	6.23 ± 0.86 *	19.01 ± 2.53	23.15 ± 4.17	24.12 ± 1.75
Tocada	2.77 ± 1.39 *	12.27 ± 2.13	3.69 ± 1.59 *	19.15 ± 2.36	18.49 ± 2.04 *	26.22 ± 1.59

Significant differences (*t*-test *, *p ≤* 0.05) between control (C) and inoculated (I) leaves for each variety and measuring day are shown, values indicate mean ± standard error (*n =* 5).

**Table 4.** Blue-to-far-red fluorescence ratio (BFRR_UV) and ‘Simple Fluorescence Ratio’ (SFR_G). The fluorescence signals were recorded from control (C), powdery mildew and leaf rust inoculated (I) leaves of the barley varieties Belana, Marthe, Conchita and Tocada at 3, 6 and 9 days after inoculation (dai).

**Fluorescence Ratio**	**Barley Variety**	**3 dai**		**6 dai**		**9 dai**	
		
		**C**	**I**	**C**	**I**	**C**	**I**
**Powdery Mildew**							

BFRR_UV	Belana	1.19 ± 0.06 *	1.60 ± 0.08	1.13 ± 0.03 *	1.76 ± 0.10	1.12 ± 0.06 *	3.42 ± 0.30
	Marthe	1.26 ± 0.11	1.23 ± 0.02	1.33 ± 0.07	1.25 ± 0.02	1.36 ± 0.06	1.28 ± 0.04
	Conchita	1.18 ± 0.04	1.23 ± 0.05	1.24 ± 0.04	1.26 ± 0.05	1.27 ± 0.03	1.23 ± 0.05
	Tocada	1.36 ± 0.03	1.63 ± 0.17	1.35 ± 0.04 *	1.87 ± 0.19	1.32 ± 0.01 *	5.21 ± 0.61
SFR_G	Belana	4.27 ± 0.02 *	4.74 ± 0.15	4.74 ± 0.11 *	4.31 ± 0.08	4.91 ± 0.28 *	3.58 ± 0.16
	Marthe	4.35 ± 0.20	4.39 ± 0.04	4.42 ± 0.05	4.26 ± 0.08	4.50 ± 0.05	4.31 ± 0.16
	Conchita	4.14 ± 0.17	4.61 ± 0.16	4.59 ± 0.10	4.79 ± 0.15	4.88 ± 0.26	4.42 ± 0.15
	Tocada	4.51 ± 0.25	4.79 ± 0.17	4.54 ± 0.06 *	4.19 ± 0.04	4.39 ± 0.06 *	2.97 ± 0.08

**Leaf Rust**							

BFRR_UV	Belana	1.43 ± 0.02 *	1.59 ± 0.06	1.49 ± 0.05 *	1.79 ± 0.06	1.54 ± 0.02 *	2.09 ± 0.03
	Marthe	1.33 ± 0.03	1.35 ± 0.03	1.34 ± 0.03 *	1.49 ± 0.05	1.37 ± 0.05	1.47 ± 0.13
	Conchita	1.48 ± 0.06	1.49 ± 0.04	1.49 ± 0.04	1.64 ± 0.06	1.52 ± 0.09	1.91 ± 0.18
	Tocada	1.46 ± 0.04 *	1.60 ± 0.03	1.53 ± 0.04 *	1.75 ± 0.07	1.52 ± 0.02 *	2.03 ± 0.10
SFR_G	Belana	4.56 ± 0.07 *	4.23 ± 0.04	4.89 ± 0.15 *	4.19 ± 0.13	4.61 ± 0.14 *	3.73 ± 0.18
	Marthe	4.30 ± 0.07 *	3.88 ± 0.12	4.61 ± 0.10 *	4.10 ± 0.16	4.31 ± 0.06 *	3.57 ± 0.17
	Conchita	4.29 ± 0.12	4.11 ± 0.08	4.62 ± 0.13	4.37 ± 0.16	4.38 ± 0.13	3.90 ± 0.19
	Tocada	4.27 ± 0.07	4.32 ± 0.15	4.79 ± 0.08 *	4.18 ± 0.19	4.36 ± 0.13 *	3.67 ± 0.12

Significant differences (*t*-test *, *p ≤* 0.05) between control (C) and inoculated (I) leaves for each variety and measuring day are shown, values indicate mean ± standard error (*n =* 5).
